# Noninvasive Anatomical and Functional Imaging for Hemodynamic Relevance in Right Coronary Artery Anomalies

**DOI:** 10.1001/jamacardio.2025.2993

**Published:** 2025-09-10

**Authors:** Marius R. Bigler, Anselm W. Stark, Federico Caobelli, Axel Rominger, Ryota Kakizaki, Flavio G. Biccirè, Saddam M. A. Al-Sabri, Isaac Shiri, Matthias Siepe, Stephan Windecker, Lorenz Räber, Christoph Gräni

**Affiliations:** 1 Department of Cardiology, Inselspital University Hospital of Bern, University of Bern, Bern, Switzerland; 2 University Clinic of Nuclear Medicine, Inselspital University Hospital of Bern, Bern, Switzerland; 3 Centre for Congenital Heart Disease, Department of Cardiovascular Surgery, Inselspital University Hospital of Bern, Bern, Switzerland; 4 Associate Editor, *JAMA Cardiology*

## Abstract

**Importance:**

Right anomalous aortic origin of a coronary artery (R-AAOCA) is a rare congenital condition increasingly diagnosed with the growing use of cardiac imaging. Due to dynamic compression of the anomalous vessel, invasive fractional flow reserve (FFR) during a dobutamine-atropine volume challenge (FFR-dobutamine) is considered the reference standard. A reliable alternative method is needed to reduce extensive invasive testing, but it remains uncertain whether noninvasive imaging can accurately assess the hemodynamic relevance of R-AAOCA.

**Objective:**

To evaluate the diagnostic performance of noninvasive anatomical and functional cardiac imaging to determine the hemodynamic relevance of R-AAOCA compared with the FFR-dobutamine reference standard.

**Design, Setting, and Participants:**

This was a prospective, single-center cohort study performed between June 2020 and January 2025. The study was conducted at a specialized coronary artery anomaly clinic in Bern, Switzerland. Consecutive adult patients with R-AAOCA with an interarterial/intramural course and a right coronary dominance were included in the study.

**Interventions:**

All patients underwent coronary computed tomography angiography (CCTA), nuclear cardiac imaging, and invasive FFR-dobutamine testing.

**Main Outcomes and Measures:**

Hemodynamic relevance of the anomalous vessel was defined as an FFR-dobutamine value less than or equal to 0.8. Patients with stenotic atherosclerotic plaques in the anomalous vessel at the time of functional testing were excluded.

**Results:**

A total of 55 patients (mean [SD] age, 51 [12] years; 37 male [67%]) with newly detected R-AAOCA and combined interarterial/intramural course were included in the analysis. Median FFR-dobutamine was 0.87 (IQR, 0.80-0.91), and 15 cases (27%) were hemodynamically relevant (ie, FFR-dobutamine ≤0.8). Anatomical CCTA (ie, CCTA–ostial minor axis) assessment demonstrated both a 100% sensitivity and negative predictive value with a receiver operating characteristic curve of 0.82, as well as a specificity of 57%, leading to rule out 23 cases (42%; ie, 58% of the hemodynamic nonrelevant cases). Functional nuclear imaging detected ischemia in 4 patients (7%; ie, 27% of hemodynamically relevant cases, all true positive, none false positive), resulting in a sensitivity of 27%, both specificity and positive predictive value of 100%, and an accuracy of 80% in predicting FFR-dobutamine less than or equal to 0.8.

**Conclusions and Relevance:**

Results of this cohort study suggest that in adults with R-AAOCA, a multimodality diagnostic imaging approach applicable in a stepwise manner, starting with CCTA, which offers high diagnostic performance to exclude hemodynamic relevance—and optionally complemented by functional imaging with modest diagnostic performance to rule in hemodynamic relevance—may help to reduce the need for invasive testing to a subset of patients.

## Key Points

**Question:**

Can noninvasive anatomical and functional imaging accurately assess the hemodynamic relevance of right anomalous aortic origin of a coronary artery (R-AAOCA)?

**Findings:**

In this cohort study including 55 patients with R-AAOCA and interarterial/intermural course, coronary computed tomography angiography (CCTA) offered high sensitivity, and functional nuclear imaging demonstrated high specificity in predicting fractional flow reserve during dobutamine stress testing.

**Meaning:**

Results suggest that stepwise application of a noninvasive imaging approach using first CCTA followed by optional nuclear cardiac imaging may reduce the need for invasive testing in R-AAOCA to a subset of patients.

## Teaser

This cohort study investigates if noninvasive anatomical and functional imaging can accurately assess the hemodynamic relevance of right anomalous aortic origin of a coronary artery.

## Introduction

Right anomalous aortic origin of a coronary artery (R-AAOCA) is a rare congenital anomaly increasingly diagnosed due to expansion of noninvasive cardiac imaging, particularly in the assessment of suspected coronary artery disease (CAD) among middle-aged and older individuals according to US and European guidelines.^1,2^ Although the majority of these anomalies are clinically insignificant,^3^ a specific concern arises with the subgroup of R-AAOCA featuring an interarterial course (between the aorta and pulmonary artery), combined with an intramural segment (ie, course within the tunica media of the aortic wall). This anatomical configuration has been associated with an increased risk of ischemia and sudden cardiac death.^4^ However, the mere presence of these anatomical features does not necessarily indicate hemodynamic relevance or warrant revascularization, making it challenging to identify those at elevated risk. Moreover, therapeutic management decisions should not solely rely on anatomical considerations of the anomaly itself^5,6,7^ because, unlike stenosis from CAD, myocardial ischemia in R-AAOCA can result from variable nonatherosclerotic parameters of fixed and dynamic stenotic components.^8^ Both European^9^ and US guidelines^10^ emphasize the importance of functional assessment in the clinical setting of R-AAOCA, particularly using an effective method for a comprehensive assessment of fixed and dynamic components to reduce false-negative results. This typically involves invasive coronary angiography with pharmacological stress testing, such as fractional flow reserve (FFR) during a dobutamine-atropine volume challenge (FFR-dobutamine).^8^ This procedure requires substantial expertise and specialized equipment, and it may cause patient discomfort. Further, intubation failure can occur, and it carries a small but nonnegligible risk, highlighting the unmet need for a noninvasive imaging approach in this clinical setting.

To address these gaps, we aimed to systematically compare the diagnostic performance of noninvasive anatomical coronary computed tomography angiography (CCTA) and functional nuclear imaging against the invasive FFR-dobutamine reference standard.

## Methods

This cohort study was approved by the ethics committee of the Canton of Bern, Switzerland (KEK 2020-00841) and complies with the Declaration of Helsinki. Consecutive adult patients with newly detected R-AAOCA and presence of combined interarterial and intramural course presenting at our specialized coronary artery anomaly clinic between June 2020 and January 2025 were prospectively enrolled. Inclusion criteria were age older than 18 years, right coronary dominance, and provision of written informed consent. This study followed the Strengthening the Reporting of Observational Studies in Epidemiology (STROBE) reporting guidelines.

For this study, all patients underwent noninvasive multimodality cardiac imaging by CCTA, physical stress single photon emission computed tomography (SPECT), or dobutamine (ie, to mimic physical exercise) stress positron emission tomography (PET) nuclear imaging as well as the invasive coronary angiography FFR-dobutamine reference standard. A detailed description of the diagnostic management has been published before^11^ and is further outlined in the eMethods and eFigure 1 in Supplement 1. Information on race and ethnicity was not routinely gathered for this study.

### Statistical Analysis

Hemodynamic relevance was defined by an FFR-dobutamine less than or equal to 0.8.^8^ In patients with confirmed hemodynamic relevance, treatment strategy (ie, surgical unroofing) was determined through interdisciplinary evaluation based on test results, symptoms, individual risk factors, and patient preference. The analysis of treatment decisions is beyond the scope of the current study. Normally distributed continuous variables were presented as mean (SD), and nonnormally distributed variables were reported as median (IQR).

## Results

Among 109 consecutive patients newly diagnosed with R-AAOCA, 55 patients were enrolled for the current study (eFigure 2 in Supplement 1). The mean (SD) age was 51 (12) years, 18 patients were female (33%), and 37 patients were male (67%). Baseline characteristics are shown in the Table. None of the patients had stenotic atherosclerotic plaques within the anomalous vessel at time of functional testing. However, 7 patients (13%) had concomitant CAD, including 2 (4%) with stenoses greater than 50% in the anomalous vessel distal to its course. These lesions were all treated with stenting before the noninvasive and invasive functional diagnostic evaluation. Median FFR-dobutamine was 0.87 (IQR, 0.80-0.91; range, 0.45-1.0), and 15 cases (27%) were hemodynamically relevant (ie, FFR-dobutamine ≤0.8). Of note, 9 of 15 cases showed an FFR-dobutamine value between 0.75 and 0.80.

**Table.** Baseline Characteristics and Diagnostics^a^

Variable	All, No. (%) (N = 55)	FFR, No. (%)		*P* value
		>0.8 (n = 40)	≤0.8 (n = 15)	
Age, mean (SD), y	51 (12)	53 (12)	48 (13)	.66
Sex				
Female	18 (33)	11 (28)	7 (47)	.74
Male	37 (67)	29 (72)	8 (53)	
BMI, mean (SD)^b^	26.6 (4.6)	27.2 (4.5)	24.8 (4.5)	.33
History of dyslipidemia	28 (51)	21 (51)	7 (47)	>.99
History of diabetes	2 (4)	2 (4)	0	NA
History of tobacco	23 (42)	16 (40)	7 (47)	>.99
History of hypertension	16 (29)	12 (30)	4 (27)	>.99
Family history of CAD	17 (31)	12 (30)	5 (33)	>.99
Family history of SCD	4 (7)	4 (10)	0	NA
Asymptomatic	8 (15)	6 (15)	2 (13)	>.99
Typical angina pectoris	31 (56)	23 (57)	8 (53)	>.99
Atypical angina pectoris	21 (38)	17 (42)	4 (27)	.47
Dyspnea	15 (27)	10 (25)	5 (33)	>.99
Syncope	5 (9)	2 (4)	3 (20)	.62
Atherosclerosis	7 (13)	5 (13)	2 (13)	>.99
Prior testing, treated CAD ≥50% stenosis in anomalous vessel	2 (4)	1 (2)	1 (7)	NA
Coronary dominance				
Right	51 (93)	38 (95)	13 (87)	.63
Balanced	4 (7)	2 (5)	2 (13)	
Left	0 (2)	0	0	
Positive nuclear stress testing	4 (7)	0	4 (7)	NA
CCTA-MLA, median (IQR), mm^2^	5.2 (4.3-6.9)	6.0 (4.66-7.30)	4.1 (3.0-4.78)	<.001
CCTA MLA-minor axis, mean (SD), mm	1.7 (0.4)	1.8 (0.4)	1.3 (0.3)	<.001
CCTA ostial area, mean (SD), mm^2^	6.9 (2.4)	7.8 (2)	4.7 (1.8)	<.001
CCTA ostial minor axis, mean (SD), mm	1.7 (0.5)	1.9 (0.4)	1.3 (0.2)	<.001
FFR-dobutamine, median (IQR)	0.87 (0.80-0.91)	0.89 (0.87-0.92)	0.76 (0.66-0.8)	<.001
Underwent stenting	2 (4)	0	2 (13)	NA
Underwent surgery	11 (20)	0	11 (73)	NA
Sports restriction	7 (13)	4 (10)	3 (20)	.80
Start aspirin	14 (24)	5 (13)	9 (60)	.007
Start β-blocker	5 (9)	1 (2)	4 (27)	.65
Start statin	11 (19)	7 (17)	4 (27)	>.99

Abbreviations: BMI, body mass index; CAD, coronary artery disease; CCTA, coronary computed tomography angiography; FFR, fractional flow reserve; FFR-dobutamine, fractional flow reserve during a dobutamine-atropine volume challenge; MLA, minimal lumen area; NA, not applicable; OLA, ostial lumen area; SCD, sudden cardiac death.

^a^ Percentage figures relative to the investigations conducted, rather than to the entire patient population.

^b^ Calculated as weight in kilograms divided by height in meters squared.

### CCTA-Derived Anatomical Features to Predict Hemodynamic Relevance

In the logistic regression analysis, CCTA minimal lumen area (odds ratio [OR], 0.27; CI, 0.09-0.54; *P* = .002), CCTA minimal lumen area minor axis (OR, 0.002; CI, 0-0.04; *P* = .001), CCTA ostial lumen area (OR, 0.51; CI, 0.32-0.74; *P* = .002), and CCTA ostial minor axis (OR, 0.01; CI, 0-0.13; *P* = .002) were most predictive for invasively detected hemodynamic relevance (eTable 1 in Supplement 1 presents other anatomical features). For receiver operating characteristic curve analysis, cutoffs of the variables were chosen to maximize sensitivity. The best diagnostic performance (including an intrarater/interrater reproducibility analysis in eTable 2 in Supplement 1) was achieved by CCTA ostial minor axis (area under the curve = 0.82) with a cutoff of greater than or equal to 1.8 mm, which resulted in 100% sensitivity and 100% negative predictive value (NPV) as well as 57% specificity and 47% positive predictive value (PPV) (eFigure 3 in Supplement 1). The results of the other CCTA parameters as well as the complete intrarater/interrater reproducibility analysis are shown in the eResults, eFigure 4, and eTable 3 in Supplement 1.

### Hemodynamic Changes During Stress Testing

There were no significant differences in heart rate, systolic aortic pressure, diastolic aortic pressure, or mean aortic pressure between stress test during invasive hemodynamic and the noninvasive functional assessment (eTable 4 in Supplement 1).

### Nuclear Stress Testing Results

Nuclear imaging either during physical stress SPECT (n = 26) or during dobutamine PET (n = 29) resulted in an accuracy of 80% with 100% specificity, 100% PPV, 27% sensitivity, and 78% NPV in predicting FFR-dobutamine less than or equal to 0.8. This allowed identification of 4 true-positive cases with 0 false-positive cases (eFigure 5 in Supplement 1 displays the confusion matrices).

**Figure 1.**
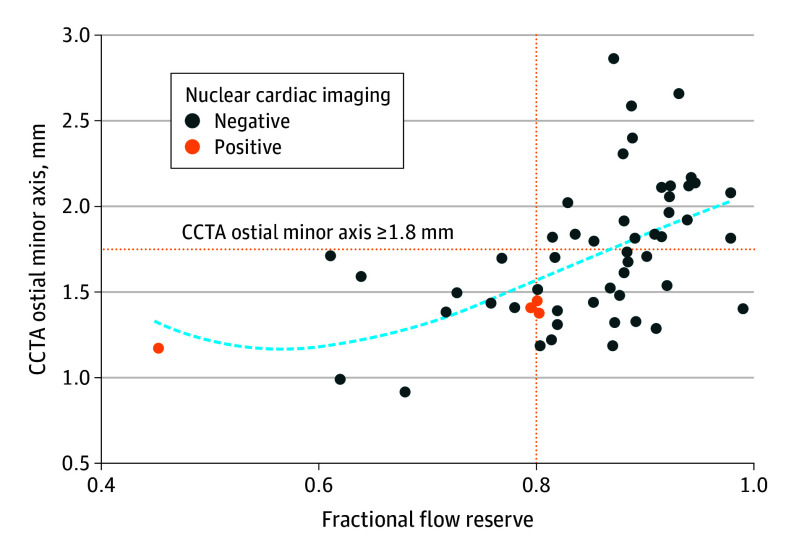
Scatterplot of Fractional Flow Reserve (FFR) During a Dobutamine-Atropine Volume Challenge, Coronary Computed Tomography Angiography (CCTA) Ostial Minor Axis, and Nuclear Cardiac Imaging Result The curve represents a second-order polynomial fitted to the values. The vertical orange dotted line represents the threshold for FFR. The horizontal orange dotted line represents the threshold for CCTA ostial minor axis. The curved blue dotted line is the fitted Loess function.

**Figure 2.**
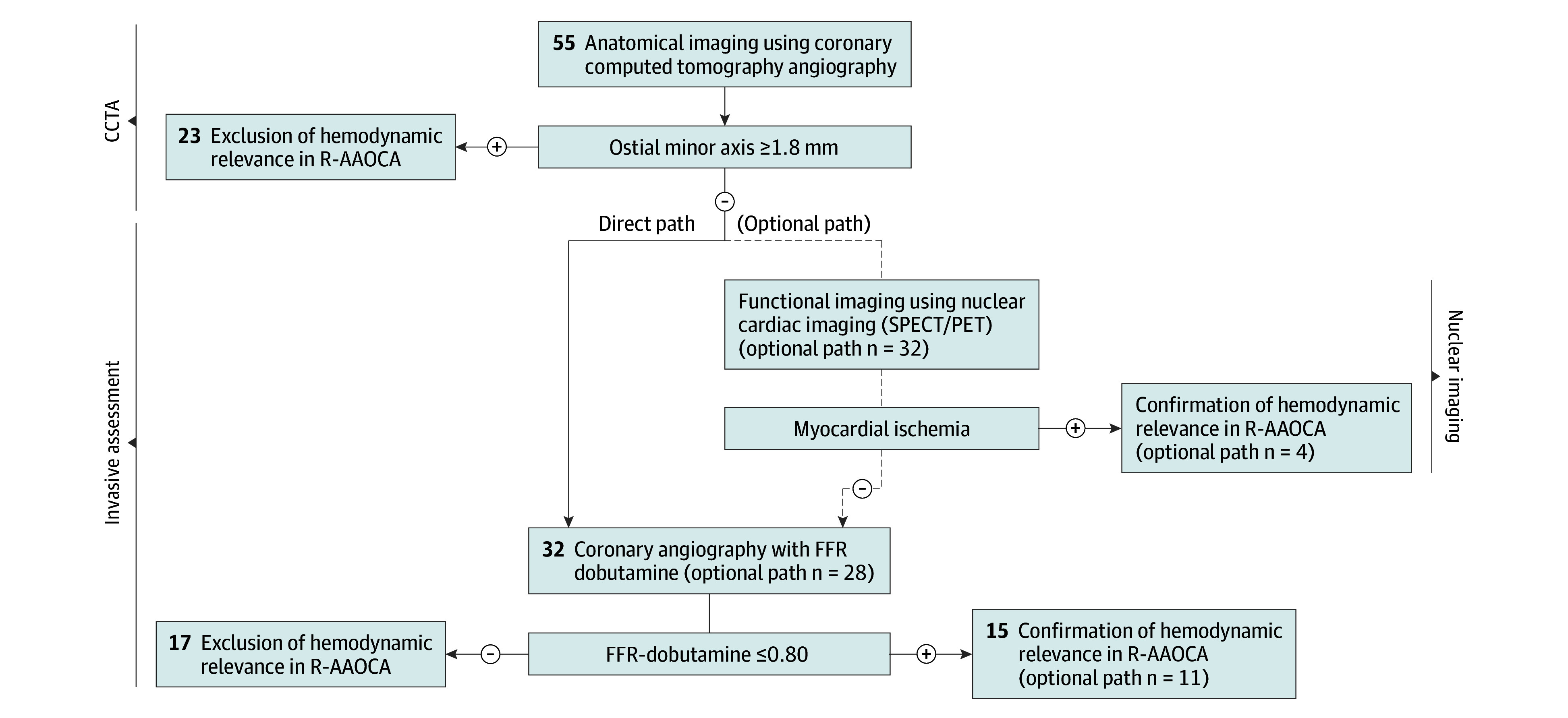
Possible Stepwise Diagnostic Approach for the Noninvasive Assessment of Hemodynamic Relevance in Right Anomalous Aortic Origin of a Coronary Artery (R-AAOCA) With Interarterial/Intramural Course FFR-dobutamine indicates fractional flow reserve during a dobutamine-atropine volume challenge; PET, positron emission tomography under dobutamine (ie, to mimic physical exercise); SPECT, single photon emission computed tomography during physical exercise.

By using a multimodality cardiac imaging approach combining anatomical and functional noninvasive imaging, a total of 27 patients (49%) were correctly assessed (Figure 1). Thereof, 23 patients (42%) with a hemodynamic nonrelevant R-AAOCA were ruled out by CCTA (ie, 58% of the hemodynamic nonrelevant cases), and 4 patients (7%) with a hemodynamic relevant R-AAOCA according to FFR-dobutamine testing were correctly ruled in by noninvasive functional imaging. Figure 2 provides a possible stepwise diagnostic approach, and eFigure 6 in Supplement 1 contains illustrative cases.

## Discussion

In this prospective cohort study, we assessed the diagnostic performance of noninvasive consecutive anatomical and functional imaging for hemodynamic relevance in R-AAOCA, using the largest cohort to date, to our knowledge, and comparing it with the FFR-dobutamine reference standard. Study results suggest that this combined approach allowed CCTA to rule out hemodynamic relevance and functional cardiac imaging to rule it in, thereby reducing the need for invasive testing to a subset of patients with R-AAOCA.

### CCTA Anatomical Assessment

CCTA was able to rule out hemodynamic relevant AAOCA compared with abnormal FFR-dobutamine results with a high sensitivity using various anatomical parameters. It is noteworthy that the minor axis demonstrated the best diagnostic performance, as this characteristic is particularly relevant in AAOCA, where the ostium and proximal intramural course typically exhibit concomitant vessel deformation (ie, elliptical vessel shape). Although there is a paucity of data applying these parameters to a population with AAOCA, the concept of CCTA minor axis (ie, minimal lumen diameter for CAD) has been tested and validated for the hemodynamic assessment of atherosclerotic lesions.^12^ Furthermore, CCTA has been compared with intravascular imaging and demonstrated a high correlation, although CCTA tended to overestimate lesion severity.^13^ However, this overestimation is typically due to calcification, an issue that does not arise in the intramural section of AAOCA. Moreover, overestimation of stenosis severity actually enhances the safety of this approach, whereas diagnostic inaccuracies in small vessel sizes do not diminish the overall effectiveness of CCTA. This observation is supported by evidence that the diagnostic accuracy of CCTA diminishes in vessels with a minimal lumen diameter less than 1 mm,^14^ and it is therefore limited in ruling in hemodynamic relevance. Hence, CCTA is only useful in larger diameters to rule out hemodynamic relevance,^15^ ie, it can be considered the D-dimer test of AAOCA.

### Noninvasive Functional Assessment

Using a prespecified anomaly testing protocol, we systematically compared nuclear imaging with FFR-dobutamine in patients with R-AAOCA without stenotic plaques.^11^ Noninvasive functional assessment identified only one-third of hemodynamically significant cases, with half of the false negatives in the FFR gray zone. Misclassification may stem from factors beyond anatomy and stress testing, including volume status, exercise type, and nonsimultaneous testing. Furthermore, given that 87% of patients did not show any atherosclerotic lesions, a healthy coronary microcirculation can be assumed allowing functional coronary autoregulation to maintain adequate blood flow across a wider range. As a result, the extent of myocardial ischemia tends to be less pronounced, and the likelihood of myocardial ischemia being confined to the subendocardial region (ie, below the detection threshold for nuclear cardiac imaging) increases.

Based on these findings and considering that no patient excluded by CCTA showed a positive result on functional imaging, a stepwise multimodality approach may be advisable for assessing hemodynamic relevance in R-AAOCA using CCTA (a sensitive test with high diagnostic performance) first to exclude hemodynamic relevance, followed by invasive testing in the remaining unclear cases. To further reduce invasive testing to a smaller subset of patients, functional testing (a specific test with modest diagnostic performance) may be included as an optional intermediate step in the remaining cases to rule in hemodynamic relevance (Figure 2). This approach, although requiring prospective validation and cost-effectiveness analysis, could further simplify diagnostic management in nonspecialized clinical settings.

### Limitations

This study has some limitations. First, this study was carried out at a single center enrolling adult patients and focusing exclusively on R-AAOCA, which is one of the most common types of AAOCA. This targeted approach enhances our understanding of R-AAOCA, although it may limit the broader applicability of our findings to other coronary anomalies, particularly concerning left coronary dominance, left AAOCA, intraseptal course, and pediatric populations. Furthermore, the rarity of AAOCA restricted the sample size available for internal validation, which limits the robustness of our findings, as well as external validation is currently lacking. Second, we defined hemodynamic relevance using an FFR-dobutamine value less than or equal to 0.8, aligning with the established cutoff for CAD evaluation. This approach represents the best currently available method, despite the absence of specific evidence confirming that the unique pathomechanisms in AAOCA can be evaluated using the same criteria as those for CAD. In CAD, FFR is not only an indicator of myocardial ischemia but also of the atherosclerotic burden, ie, the most relevant substrate of cardiac events, unlike AAOCA, where atherosclerosis is absent and events may be only related to ischemia related arrhythmias. Nevertheless, despite the differences in disease mechanisms between CAD and AAOCA, considering the documented beneficial outcomes in patients with CAD and an FFR above the threshold, the likelihood of experiencing a major cardiac event in patients with AAOCA is reasonably low. Third, as no patient in our cohort showed atherosclerotic CAD stenosis within the anomalous vessels before functional testing, the need for further clarification on how to address with overlapping diseases is evident.

## Conclusions

In this cohort study of adults with R-AAOCA, results suggest that a multimodality diagnostic imaging approach applicable in a stepwise manner, starting with CCTA, which offers high diagnostic performance to exclude hemodynamic relevance—and optionally complemented by functional imaging with modest diagnostic performance to rule in hemodynamic relevance—may help to reduce the need for invasive testing to a subset of patients.

## Notes

Supplement 1.
eMethods.

eResults.
**eFigure 1.** Assessment of the Anatomical High-Risk Features by Coronary Computed Tomography Angiography Including a Step-by-Step Instruction **eFigure 2.** Flowchart of the Study Recruitment **eFigure 3.** Receiver-Operating Characteristics of Coronary Computed Tomography Angiography (CCTA) Ostial Minor Axis Using FFR-Dobutamine ≤/>0.80 as Reference **eFigure 4.** Bland and Altman Plot for Intrarater and Interrater Variability of CCTA Ostial Minor Axis **eFigure 5.** Confusion Matrices of the Noninvasive Cardiac Imaging Modalities **eFigure 6.** Illustration of 3 Different Cases **eTable 1.** Logistic Regression Results of the CCTA Anatomical Features **eTable 2.** Intrarater and Interrater Intraclass Correlation Coefficients (ICC) of the Different Anatomical Parameters Assessed by Coronary Computed Tomography Angiography With 95% CI **eTable 3.** Performance Metrics of the Coronary Computed Tomography Angiography in Detecting Hemodynamic Relevant Coronary Anomalies According to FFR-Dobutamine **eTable 4.** Hemodynamic Changes During Stress Testing
eReferences

Supplement 2.
Data Sharing Statement.
