# Supplementary material for: Noninvasive Anatomical and Functional Imaging for Hemodynamic Relevance in Right Coronary Artery Anomalies
Source: JAMA Cardiol. 2025 Sep 10;10(10):1055–60. doi: 10.1001/jamacardio.2025.2993 (PMC12423952; doi:10.1001/jamacardio.2025.2993)
Supplement: Supplement 1. — eMethods. eResults. eFigure 1. Assessment of the Anatomical High-Risk Features by Coronary Computed Tomography Angiography Including a Step-by-Step Instruction eFigure 2. Flowchart of the Study Recruitment eFigure 3. Receiver-Operating Characteristics of Coronary Computed Tomography Angiography (CCTA) Ostial Minor Axis Using FFR-Dobutamine ≤/>0.80 as Reference eFigure 4. Bland and Altman Plot for Intrarater and Interrater Variability of CCTA Ostial Minor Axis eFigure 5. Confusion Matrices of the Noninvasive Cardiac Imaging Modalities eFigure 6. Illustration of 3 Different Cases eTable 1. Logistic Regression Results of the CCTA Anatomical Features eTable 2. Intrarater and Interrater Intraclass Correlation Coefficients (ICC) of the Different Anatomical Parameters Assessed by Coronary Computed Tomography Angiography With 95% CI eTable 3. Performance Metrics of the Coronary Computed Tomography Angiography in Detecting Hemodynamic Relevant Coronary Anomalies According to FFR-Dobutamine eTable 4. Hemodynamic Changes During Stress Testing eReferences [file jamacardiol-e252993-s001.pdf]

Supplementary Online Content

Bigler MR, Stark AW, Caobelli F, et al. Noninvasive anatomical and functional imaging for hemodynamic relevance in right coronary artery anomalies. *JAMA Cardiol*. Published online September 10, 2025. doi:10.1001/jamacardio.2025.2993

eMethods.

eResults.

eFigure 1. Assessment of the Anatomical High-Risk Features by Coronary Computed Tomography Angiography Including a Step-by-Step Instruction

eFigure 2. Flowchart of the Study Recruitment

eFigure 3. Receiver-Operating Characteristics of Coronary Computed Tomography Angiography (CCTA) Ostial Minor Axis Using FFR-Dobutamine ≤/>0.80 as Reference

eFigure 4. Bland and Altman Plot for Intrarater and Interrater Variability of CCTA Ostial Minor Axis

eFigure 5. Confusion Matrices of the Noninvasive Cardiac Imaging Modalities

eFigure 6. Illustration of 3 Different Cases

eTable 1. Logistic Regression Results of the CCTA Anatomical Features

eTable 2. Intrarater and Interrater Intraclass Correlation Coefficients (ICC) of the Different Anatomical Parameters Assessed by Coronary Computed Tomography Angiography With 95% CI

eTable 3. Performance Metrics of the Coronary Computed Tomography Angiography in Detecting Hemodynamic Relevant Coronary Anomalies According to FFR-Dobutamine

eTable 4. Hemodynamic Changes During Stress Testing

eReferences

This supplementary material has been provided by the authors to give readers additional information about their work.

© 2025 Bigler MR et al. *JAMA Cardiology*.

eMethods

Study population

Consecutive adult (age ≥18 years) patients with newly detected R-AAOCA and presence of interarterial and intramural course presenting at our specialized coronary artery anomaly clinic between 06/2020 and 01/2025 were prospectively enrolled. Inclusion criteria were age>18years, as well as provision of written informed consent. For this study, all patients underwent noninvasive multimodality cardiac imaging by CCTA and physical stress Single Photon Emission Computed Tomography (SPECT) or dobutamine (i.e. to mimic physical exercise) stress Positron Emission Tomography (PET) nuclear imaging[1] as well as invasive coronary angiography $FFR_{Dobutamine}$ reference standard. Local ethics approval was available (KEK 2020-00841) and the study was registered with ClinicalTrials.gov ID: NCT04475289[1].

Noninvasive anatomical assessment

Anatomical CCTA features were assessed using clinically certified software Syngo.via (Siemens, Berlin, Germany) and categorized as following[2] (eFigure 1):

- **Ostium:** Ostial lumen area (CCTA-OLA), major axis, ostial minor axis and its ratio (i.e. elliptic-ratio). Dichotomized criterion was: presence/absence of a slit-like ostium (defined as minor axis/major axis < 50%)[3]
- **Intramural course:** Minimal lumen area (CCTA-MLA) (~2mm distal to the CCTA-OLA), major axis, minor axis and its ratio (i.e. elliptic-ratio). Dichotomized criterion was: presence/absence of an elliptic vessel shape (defined as a ratio > 1.3)[4].
- **Intramural length:** Length of the centerline from the ostium to non-elliptic, distal first round lumen area[3, 5-7]
- **Take-off angle** in degrees, acute take-off angle defined as < 45°[3],

Noninvasive functional assessment

From 06/2020 to 02/2023, noninvasive functional assessment was performed by SPECT. Patients underwent a one-day ^{99m}Tc-tetrofosmin stress/rest protocol according to the established procedural guidelines for radionuclide imaging of myocardial perfusion[8]. Briefly, physical stress testing was

© 2025 Bigler MR et al. *JAMA Cardiology*.

performed on a bicycle aiming at a heart rate of 100% of the predicted maximum age-corrected heart rate (minimum aim 85% of maximum age-corrected heart rate). ^{99m}Tc-tetrofosmin was injected at peak physical stress, while rest myocardial perfusion imaging was performed thereafter with the identical acquisition protocol after injection of a three times higher dose of ^{99m}Tc-tetrofosmin. After 02/2023, noninvasive functional assessment was performed by Rubidium (^{82}Rb-Chloride) PET due to availability at our center and improved spatial and temporal resolution. In patients undergoing ^{82}Rb-PET, stress was pharmacologically induced using dobutamine-atropine stress (i.e. administration of dobutamine started at a rate of 20 mcg/kg/min for 2 minutes, followed by an increased infusion at 40 mcg/kg/min for at least 4 minutes). Additionally, 1 mg of atropine was added to increase heart rate, ensuring it reached a minimum of 85% of the target maximum rate. Dobutamine infusion was then stopped and ^{82}Rb-Chloride was injected, starting simultaneously the dynamic image acquisition.

For both nuclear imaging modalities, regional tracer uptake was assessed using the 17-segment model and the semiquantitative scoring system of defect severity and extent, as recommended by the American Society of Nuclear Cardiology[9]. Myocardial ischemia was defined as stress-induced hypoperfusion in myocardial regions supplied by the anomalous coronary artery, in the absence of resting perfusion abnormalities.

Invasive hemodynamic reference standard assessment

Coronary angiography was performed via radial access. The procedure involved intubation with a 6 or 6.5 French guiding catheter, after which a 0.0014-inch pressure-sensing intracoronary guidewire (PressureWire™ X Guidewire, Abbott, Chicago, Illinois, United States) was calibrated and positioned distal to the interarterial/intramural segment to allow precise pressure monitoring.

For simulated exercise stress testing, the protocol involved a combined dobutamine-atropine-volume (3000 ml) challenge as previsouly described[1, 10]. During peak heart rate, stationary $FFR_{Dobutamine}$ was measured, calculated as the ratio of the mean distal pressure to the mean aortic pressure, using the pressure sensor distal to the intramural segment. Following the measurement, the pressure wire was retracted to check for any necessary corrections. Hemodynamic relevance was defined by an $FFR_{Dobutamine}$ of ≤ 0.8[5, 10, 11].

Statistical analysis

The normality of continuous data was assessed using a Shapiro-Wilk test and visual inspection of Q-Q-plots. Normally distributed continuous variables were presented as means ± standard deviations, while non-normally distributed variables were reported as medians with interquartile ranges (25th to 75th percentile). To compare variables, the Student's t-test was used for normally distributed data, and the Wilcoxon signed-rank test was employed for non-normally distributed data. Two-level factors were compared using a proportion test, while multilevel categorical factors were analyzed using the chi-square test. P-values were adjusted for multiple comparisons using the false discovery rate (FDR) method by Benjamini&Hochberg. Simple logistic regression analysis was conducted with hemodynamic relevance ($FFR_{Dobutamine}$>/≤ 0.8) as the dependent variable, and continuous CCTA anatomical features as independent variables. Significant logistic regression models for continuous variables were further evaluated using receiver operating characteristics (ROC) and area under the curve (AUC). Each ROC curve was analyzed by selecting the optimal cut-off values based on Youden's method, and maximizing sensitivity and specificity alone, where applicable. To assess the clinical utility of these thresholds, metrics such as sensitivity, specificity, positive predictive value (PPV), negative predictive value (NPV), and overall accuracy were calculated for the derived cut-offs to evaluate their discriminative performance in identifying positive and negative cases within the study population. The categorical variables (positive/negative) of the nuclear imaging results were analyzed using confusion matrices together with the same performance metrices (i.e. sensitivity, specificity, PPV, NPV and accuracy). Measurement variability was assessed through both Bland-Altman analysis and intraclass correlation coefficients (ICC). The intra-rater ICC was calculated using an absolute agreement, two-way mixed-effects model, while the inter-rater ICC was calculated using an absolute agreement, two-way random-effects model. All statistical analyses were conducted using R (version 4.2.3) and the pROC, yardstick, caret, psych and irr libraries.

© 2025 Bigler MR et al. *JAMA Cardiology*.

eResults

Intra- and interreader variability analysis of CCTA derived anatomical features

Intra-rater ICC for CCTA-MLA was 0.882, while inter-rater ICC was 0.692. CCTA-MLA minor axis (intra-rater ICC 0.799, inter-rater ICC 0.634), CCTA-OLA (intra-rater ICC 0.864, inter-rater ICC 0.747) and CCTA ostial minor axis (intra-rater ICC 0.778, inter-rater ICC 0.712; eFigure 3) demonstrated similar results and according to Koo et al.[12] a good respectively medium extent of variability.

© 2025 Bigler MR et al. *JAMA Cardiology*.

eFigure 1: Assessment of the anatomical high-risk features by coronary computed tomography angiography including a step-by-step instruction

1) Multiplanar reconstruction parallel to the aortic annulus
2) On this plane, the take-off angle is measured as the tangent to the aorta and the centerline of the ostium. Acute take-off angle is then defined as an angle below 45°.
3) The next assessments involve the measurement of the lumen parameters, i.e. lumen area and major and CCTA-minor axis at different locations. Particular focus has to be given for these measurements to be exactly perpendicular to the vessel course in two planes to ensure that the lumen cross-section in the third plane of the multiplanar reconstruction is not distorted. The ostium is then assessed at the first distinguishable lumen inside of the intramural course (in green) and its area and major (red line) and minor axis (yellow line, perpendicular to the major axis in the middle) are measured. Please note the elliptic vessel shape.
4) Within the intramural course a lumen around 2 mm distal to the ostium (i.e. minimal lumen area) is again assessed including measurement of the major (red line) and minor axis (yellow line).
5) Lastly, intramural length is assessed. In CCTA, its beginning is defined by the ostium (in green) and its endpoint with the loss of the elliptic vessel shape, i.e. the first round lumen (in blue).

Abbreviations: AAOCA = anomalous aortic origin of a coronary artery, CCTA = coronary computed tomography angiography.

© 2025 Bigler MR et al. *JAMA Cardiology*.

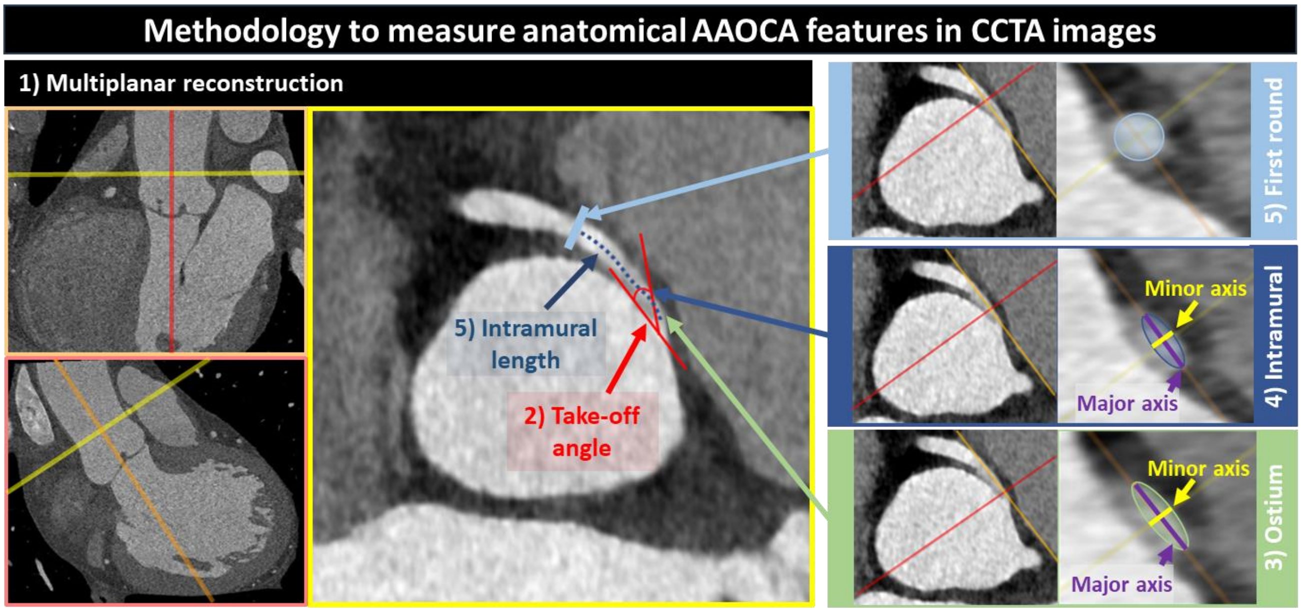

© 2025 Bigler MR et al. *JAMA Cardiology*.

eFigure 2: Flowchart of the study recruitment.

AAOCA = Anomalous aortic origin of a coronary artery, Cx-AAOCA = Circumflex anomalous aortic origin of a coronary artery, L-AAOCA = Left anomalous aortic origin of a coronary artery, R-AAOCA = Right anomalous aortic origin of a coronary artery, RCA = Right coronary artery, HOCM = Hypertrophic obstructive cardiomyopathy, IVUS = Intravascular ultrasound during resting conditions, FFR = Fractional flow reserve, FFRAdenosine = Fractional flow reserve during adenosine, $FFR_{Dobutamine}$ = Fractional flow reserve during dobutamine-atropine-volume challenge.

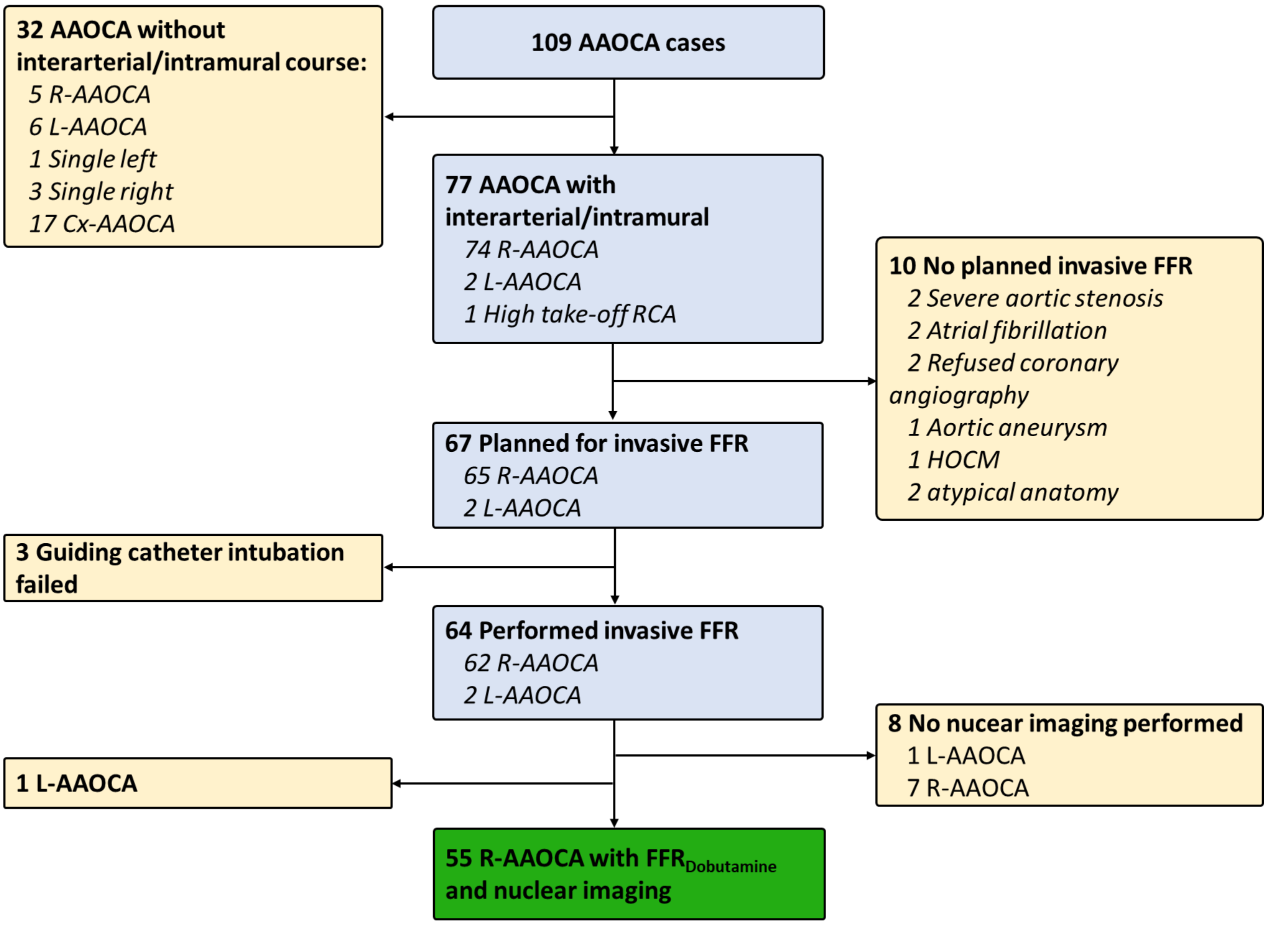

© 2025 Bigler MR et al. *JAMA Cardiology*.

eFigure 3 Receiver-operating characteristics of coronary computed tomography angiography (CCTA) ostial minor axis using $FFR_{Dobutamine}$ ≤/>0.80 as reference

AUC = area under the curve

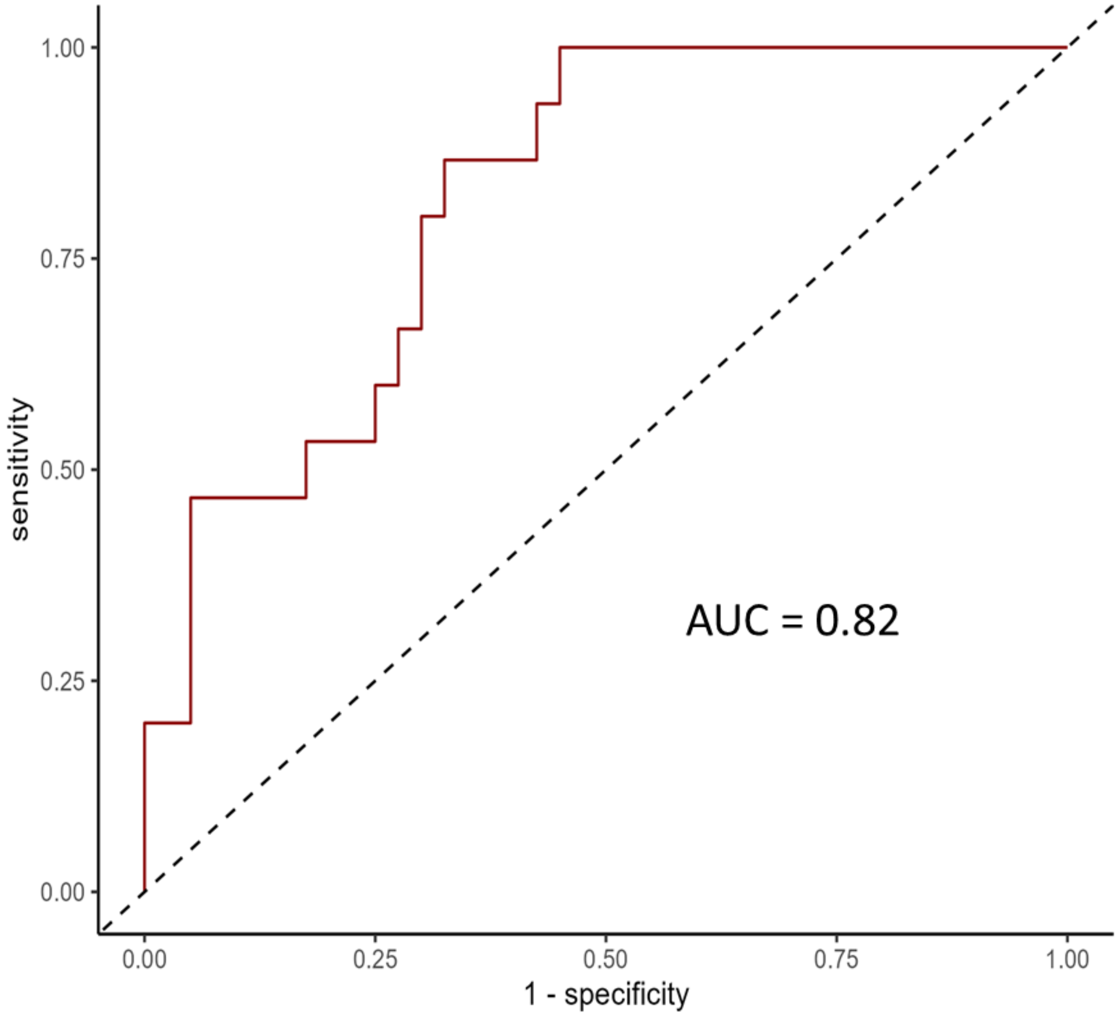

© 2025 Bigler MR et al. *JAMA Cardiology*.

eFigure 4 Bland and Altman plot for intra- and inter-rater variability of CCTA ostial minor axis.

Difference = measurement MRB minus measurement AS; Solid black lines = mean difference; dashed grey lines = 95% limits of agreement. CCTA = coronary computed tomography angiography

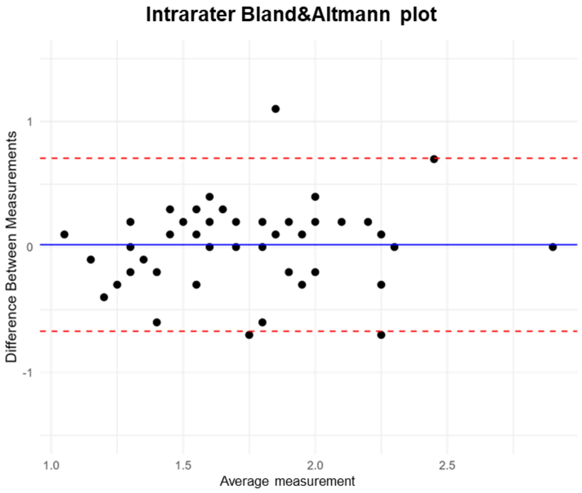

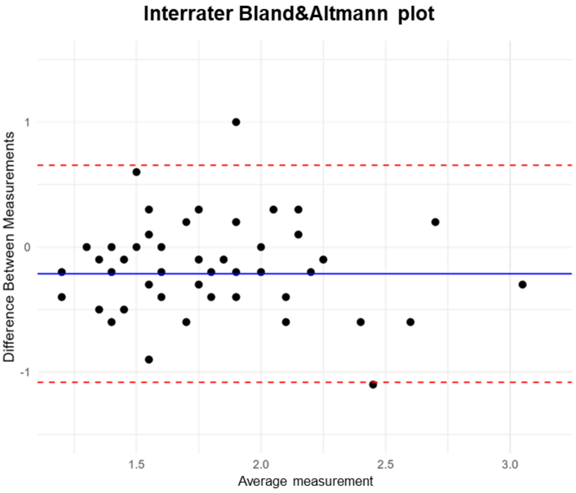

© 2025 Bigler MR et al. *JAMA Cardiology*.

eFigure 5: Confusion matrices of the noninvasive cardiac imaging modalities.

CCTA = coronary computed tomography angiography, using ostial minor axis as the diagnostic parameter. Nuclear cardiac imaging: PET = positron emission tomography (n=29). SPECT = single photon emission computer tomography (n=26)

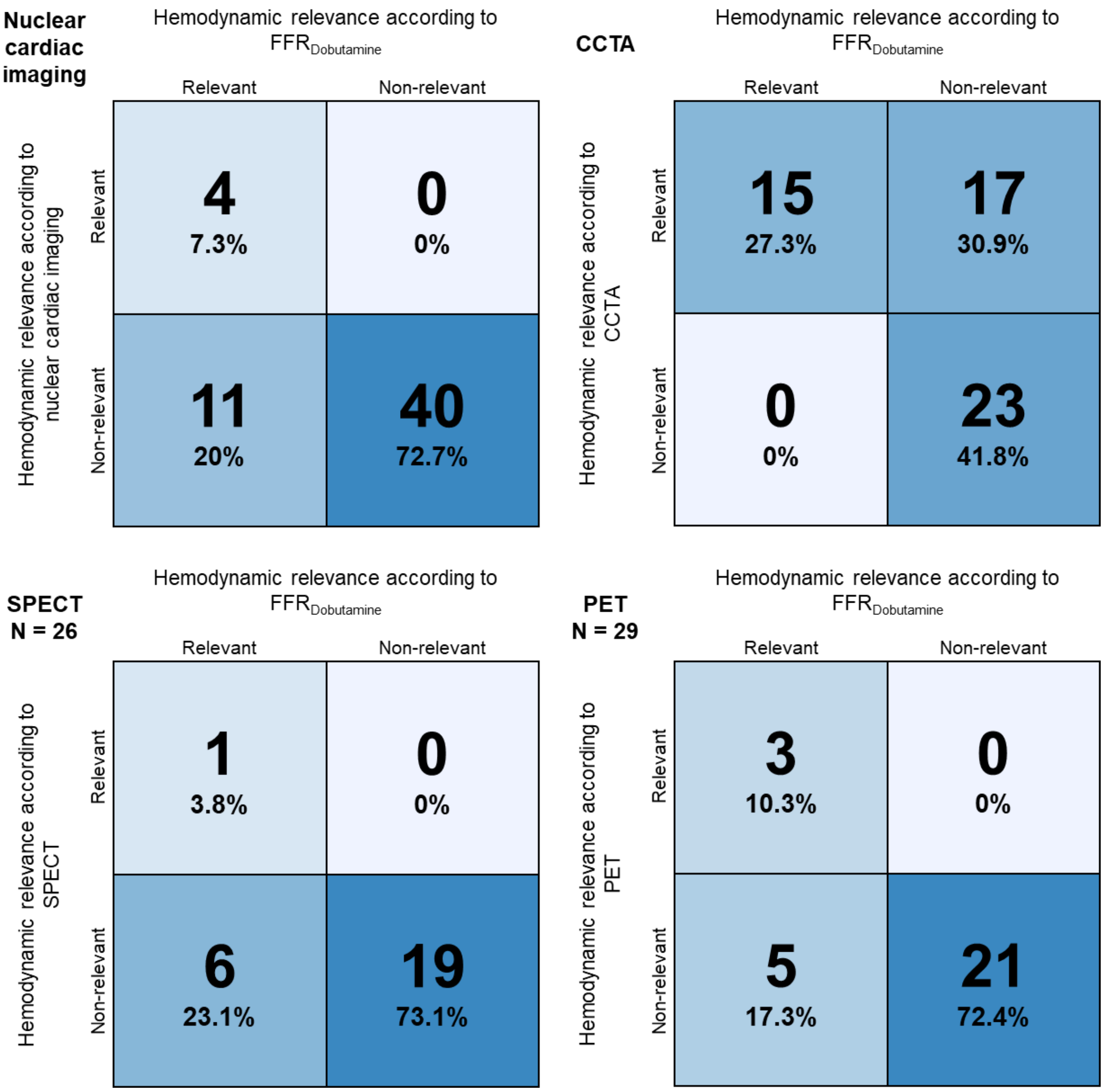

© 2025 Bigler MR et al. *JAMA Cardiology*.

eFigure 6 Illustration of three different cases

A) 41 year old male patient presenting with atypical chest pain several months after percutaneous coronary intervention of an inferior non-ST-elevation myocardial infarction due to a plaque rupture of the proximal right coronary artery. CCTA showed a R-AAOCA with intramural/interarterial course and a small CCTA- ostial minor axis, nuclear cardiac imaging demonstrated a corresponding myocardial ischemia in the territory of the anomalous coronary artery during pharmacological stress with dobutamine (maximal heart rate 162bpm, i.e. 91% of the calculated maximal heart rate). Hence, presenting a case of PET rule in. Subsequent invasive stress testing confirmed the hemodynamic relevance with a $FFR_{Dobutamine}$ of 0.80. After interdisciplinary discussion, the anomalous course was stented because of an already implanted coronary stent in the proximal (but extramural) part. **B)** 48 year old male patient with an incidental diagnosis of a R-AAOCA with origin from the left main coronary artery and intramural/interarterial course during a risk stratification due to a positive family history of myocardial infarction. The patient was free of symptoms. Based on the ostial CCTA-minor axis of 2.1mm, hemodynamic relevance could be ruled out by CCTA. This finding was confirmed by a negative nuclear cardiac imaging (maximal heart rate 140bpm, i.e. 81%) and a negative $FFR_{Dobutamine}$ of 0.91. **C)** 64 year old female patient with newly developed exertional dyspnea without angina pectoris. CCTA showed a R-AAOCA with intramural/interarterial course and a small ostial CCTA-minor axis, so that hemodynamic relevance could not be ruled out. Subsequent nuclear cardiac imaging was negative despite a sufficient pharmacologic stress test (maximal heart rate 146bpm, i.e.94%). Hence, due to unconclusive noninvasive cardiac imaging, an invasive stress test was performed demonstrating the hemodynamic relevance of this anomaly with a $FFR_{Dobutamine}$ of 0.77. After interdisciplinary discussion and based on the patients wish as well as her sedentary life style, a watchful-waiting strategy was choosen.

© 2025 Bigler MR et al. *JAMA Cardiology*.

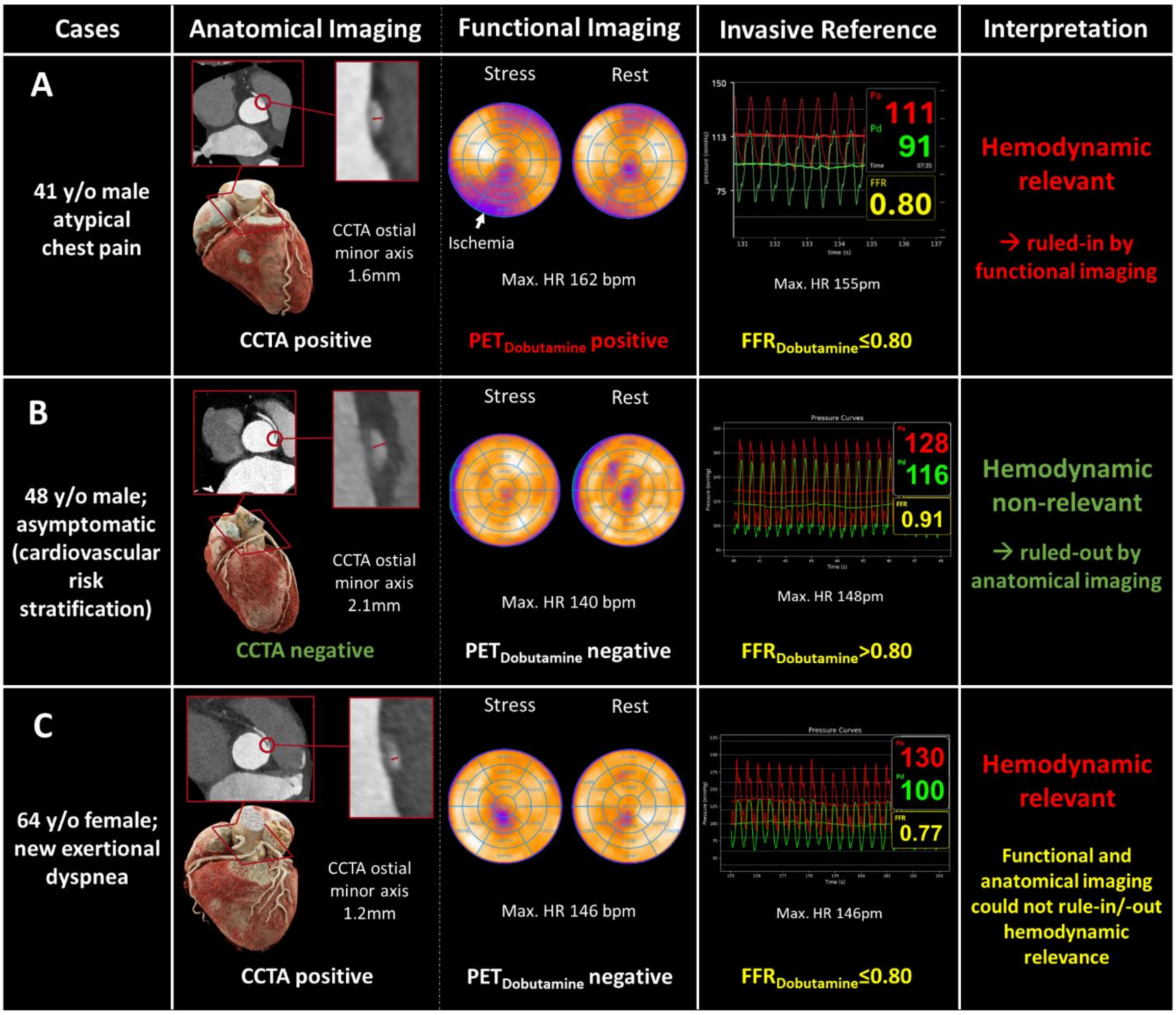

© 2025 Bigler MR et al. *JAMA Cardiology*.

eTable 1: **Logistic regression results of the CCTA anatomical features**

Variable	OR (CI)	p-value
CCTA minmal lumen area, mm^2	0.27 (0.09-0.54)	0.002
CCTA-MLA minor axis, mm	0.002 (0.00-0.04)	0.001
CCTA-MLA elliptic ratio	3.37 (1.39-9.78)	0.012
CCTA ostial lumen area, mm^2	0.51 (0.32-0.74)	0.002
CCTA ostial minor axis, mm	0.01 (0.00-0.13)	0.002
CCTA-OLA elliptic ratio	2.56 (0.94-9.28)	0.085

eTable 2: Intra-rater and inter-rater intraclass correlation coefficients (ICC) of the different anatomical parameters assessed by coronary computed tomography angiography with 95% confidence interval

Variable	Intra-rater ICC	Inter-rater ICC
CCTA minmal lumen area, mm^2	0.882 (0.856-0.903)	0.692 (0.396-0.821)
CCTA-MLA minor axis, mm	0.799 (0.755-0.835)	0.634 (0.471-0.74)
CCTA-MLA elliptic ratio	0.545 (0.445-0.627)	0 (-0.16-0.153)
CCTA ostial lumen area, mm^2	0.864 (0.834-0.888)	0.747 (0.686-0.795)
CCTA ostial minor axis, mm	0.778 (0.73-0.818)	0.712 (0.551-0.804)
CCTA-OLA elliptic ratio	0.734 (0.675-0.782)	0.599 (0.213-0.766)

© 2025 Bigler MR et al. *JAMA Cardiology*.

eTable 3: Performance metrics of the coronary computed tomography angiography in detecting hemodynamic relevant coronary anomalies according to $FFR_{Dobutamine}$.

CCTA Predictor	Cut-off value	AUC	Accuracy [%]	Sensitivity [%]	NPV [%]	Specificity [%]	PPV [%]	n true neg.	n false neg.	n false pos.	n true pos.
CCTA-MLA [mm^2]	≥5.6	0.85	73	100	100	62	50	25	0	15	15
	≥4.5		78	67	87	82	59	33	5	7	10
	≥4.2		78	53	83	88	62	35	7	5	8
	≥3.9		84	40	82	100	100	40	9	0	6
CCTA-MLA elliptic ratio	≤1.3	0.69	29	100	100	2	28	1	0	39	15
	≤2.8		78	67	87	82	59	33	5	7	10
	≤3.3		80	40	81	95	75	38	9	2	6
	≤4.1		76	13	75	100	100	40	13	0	2
CCTA-MLA minor axis [mm]	≥1.9	0.90	56	100	100	40	38	16	0	24	15
	≥1.5		76	80	91	75	55	30	3	10	12
	≥1.4		85	73	90	90	73	36	4	4	11
	≥1.3		85	47	83	100	100	40	8	0	7
CCTA-OLA [mm^2]	≥7.7	0.82	67	100	100	55	45	22	0	18	15
	≥6.3		73	80	90	70	50	28	3	12	12
	≥4.5		82	47	83	95	78	38	8	2	7
	≥3.4		78	20	77	100	100	40	12	0	3
CCTA-OLA elliptic ratio	≤1.3	0.67	29	100	100	2	28	1	0	39	15
	≤2.8		62	73	85	57	39	23	4	17	11
	≤3.1		73	60	84	78	50	31	6	9	9
	≤4.3		75	7	74	100	100	40	14	0	1
CCTA ostial minor axis [mm]	≥1.8	0.82	69	100	100	57	47	23	0	17	15
	≥1.7		73	87	93	68	50	27	2	13	13
	≥1.5		75	67	86	78	53	31	5	9	10
	≥1.1		76	13	75	100	100	40	13	0	2

© 2025 Bigler MR et al. *JAMA Cardiology*.

Abbreviations: CCTA = Coronary computed tomography angiography, $FFR_{Dobutamine}$ = Fractional flow reserve during dobutamine-atropine-volume challenge, CCTA-MLA = minimal lumen area, neg. = negative, CCTA-OLA = ostial lumen area, pos. = positive

eTable 4: Hemodynamic changes during stress testing

	**Invasive $FFR_{Dobutamine}$ N = 55**			**Nuclear imaging N = 55 (PET/CT N = 29, SPECT N = 26)**			**Change between groups***
**Variable**	**Rest**	**Stress**	**p-value (adjusted)**	**Rest**	**Stress**	**p-value (adjusted)**	**p-value (adjusted)**
Heart rate [bpm]	75 (69-84)	152 (145-158)	<0.001	70 (63-77)	146 (130-157)	<0.001	0.27
Percent of predicted maximal heart rate (220-age) [%]		90 (85-95)	N/A		87 (80-91)	N/A	0.89
Systolic aortic pressure [mmHg]	111 ± 20	142 ± 26	<0.001	117 ± 20	154 ± 29	<0.001	0.06
Diastolic aortic pressure [mmHg]	67 ± 12	72 ± 20	0.15	72 (65-81)	74 (69-79)	0.93	0.57
Mean aortic pressure [mmHg]	82 ± 14	95 ± 21	<0.001	89 ± 14	101 ± 16	0.003	0.27

Abbreviations: * = Comparison of the change (i.e. parameter @ stress minus parameter @ rest) between the two groups. Difference $FFR_{Dobutamine}$ = Fractional flow reserve during dobutamine-volume challenge, PET = positron emission tomography, SPECT = single photon emission computer tomography.

© 2025 Bigler MR et al. *JAMA Cardiology*.

eReferences

1. Bigler MR, Stark AW, Shiri I, Illi J, Siepe M, Caobelli F, Giannopoulos AA, Buechel RR, Haeberlin A, Obrist D, et al. Noninvasive anatomical assessment for ruling out hemodynamically relevant coronary artery anomalies in adults - A comparison of coronary-CT to invasive coronary angiography: The NARCO study design. *Contemp Clin Trials Commun*. 2024;42:101394.
2. Stark AW, Matthey-de-l'Endroit RL, Ferroni A, Kakizaki R, Bigler MR, Biccirè FG, Ueki Y, Haeberlin A, Siepe M, Shiri I, et al. Coronary CT Anatomy-Based Prediction of Invasively Assessed Hemodynamic Significance in Middle-Aged Patients With Right Coronary Artery Anomaly: The NARCO Study. *Circulation*. 2025;151:578-580.
3. Cheezum MK, Ghoshhajra B, Bittencourt MS, Hulten EA, Bhatt A, Mousavi N, Shah NR, Valente AM, Rybicki FJ, Steigner M, et al. Anomalous origin of the coronary artery arising from the opposite sinus: prevalence and outcomes in patients undergoing coronary CTA. *European heart journal cardiovascular Imaging*. 2017;18:224-235.
4. Harris MA, Whitehead KK, Shin DC, Keller MS, Weinberg PM and Fogel MA. Identifying Abnormal Ostial Morphology in Anomalous Aortic Origin of a Coronary Artery. *The Annals of thoracic surgery*. 2015;100:174-9.
5. Lim JC, Beale A and Ramcharitar S. Anomalous origination of a coronary artery from the opposite sinus. *Nature reviews Cardiology*. 2011;8:706-19.
6. Angelini P, Walmsley R, Cheong BY and Ott DA. Left main coronary artery originating from the proper sinus but with acute angulation and an intramural course, leading to critical stenosis. *Texas Heart Institute journal*. 2010;37:221-5.
7. Jegatheeswaran A, Devlin PJ, McCrindle BW, Williams WG, Jacobs ML, Blackstone EH, DeCampli WM, Caldarone CA, Gaynor JW, Kirklin JK, et al. Features associated with myocardial ischemia in anomalous aortic origin of a coronary artery: A Congenital Heart Surgeons' Society study. *The Journal of thoracic and cardiovascular surgery*. 2019;158:822-834.e3.
8. Verberne HJ, Acampa W, Anagnostopoulos C, Ballinger J, Bengel F, De Bondt P, Buechel RR, Cuocolo A, van Eck-Smit BL, Flotats A, et al. EANM procedural guidelines for radionuclide myocardial perfusion imaging with SPECT and SPECT/CT: 2015 revision. *Eur J Nucl Med Mol Imaging*. 2015;42:1929-40.
9. Machac J, Bacharach SL, Bateman TM, Bax JJ, Beanlands R, Bengel F, Bergmann SR, Brunken RC, Case J, Delbeke D, et al. Positron emission tomography myocardial perfusion and glucose metabolism imaging. *Journal of nuclear cardiology : official publication of the American Society of Nuclear Cardiology*. 2006;13:e121-51.
10. Bigler MR, Ashraf A, Seiler C, Praz F, Ueki Y, Windecker S, Kadner A, Räber L and Gräni C. Hemodynamic Relevance of Anomalous Coronary Arteries Originating From the Opposite Sinus of Valsalva-In Search of the Evidence. *Frontiers in Cardiovascular Medicine*. 2021;7.
11. Tonino PA, De Bruyne B, Pijls NH, Siebert U, Ikeno F, van' t Veer M, Klauss V, Manoharan G, Engstrom T, Oldroyd KG, et al. Fractional flow reserve versus angiography for guiding percutaneous coronary intervention. *The New England journal of medicine*. 2009;360:213-24.
12. Koo TK and Li MY. A Guideline of Selecting and Reporting Intraclass Correlation Coefficients for Reliability Research. *Journal of chiropractic medicine*. 2016;15:155-63.

© 2025 Bigler MR et al. *JAMA Cardiology*.
