# Supplementary material for: Noninvasive Anatomical and Functional Imaging for Hemodynamic Relevance in Right Coronary Artery Anomalies
Source: JAMA Cardiol. 2025 Sep 10;10(10):1055–60. doi: 10.1001/jamacardio.2025.2993 (PMC12423952; doi:10.1001/jamacardio.2025.2993)
Supplement: Supplement 2. — Data Sharing Statement. [file jamacardiol-e252993-s002.pdf]

Data Sharing Statement

Bigler. Noninvasive Anatomical and Functional Imaging for Hemodynamic Relevance in Right Coronary Artery Anomalies. *JAMA Cardiol*. Published September 10, 2025. doi:10.1001/jamacardio.2025.2993

Data
Data available: Yes
Data types: Deidentified participant data
How to access data: christoph.graeni@insel.ch
When available: With publication

Supporting Documents
Document types: None

Additional Information
Who can access the data: The data will be made available from the corresponding author upon reasonable request.
Types of analyses: For non-profit reasearch only
Mechanisms of data availability: After approval of a proposal
